# Supplementary material for: Acid Sphingomyelinase Deficiency Ameliorates Farber Disease
Source: Int J Mol Sci. 2019 Dec 11;20(24):6253. doi: 10.3390/ijms20246253 (PMC6941101; doi:10.3390/ijms20246253)
Supplement: Supplementary file 1 [file ijms-20-06253-s001.zip › ijms-637307-supplementary/suppl figure 1.pdf]

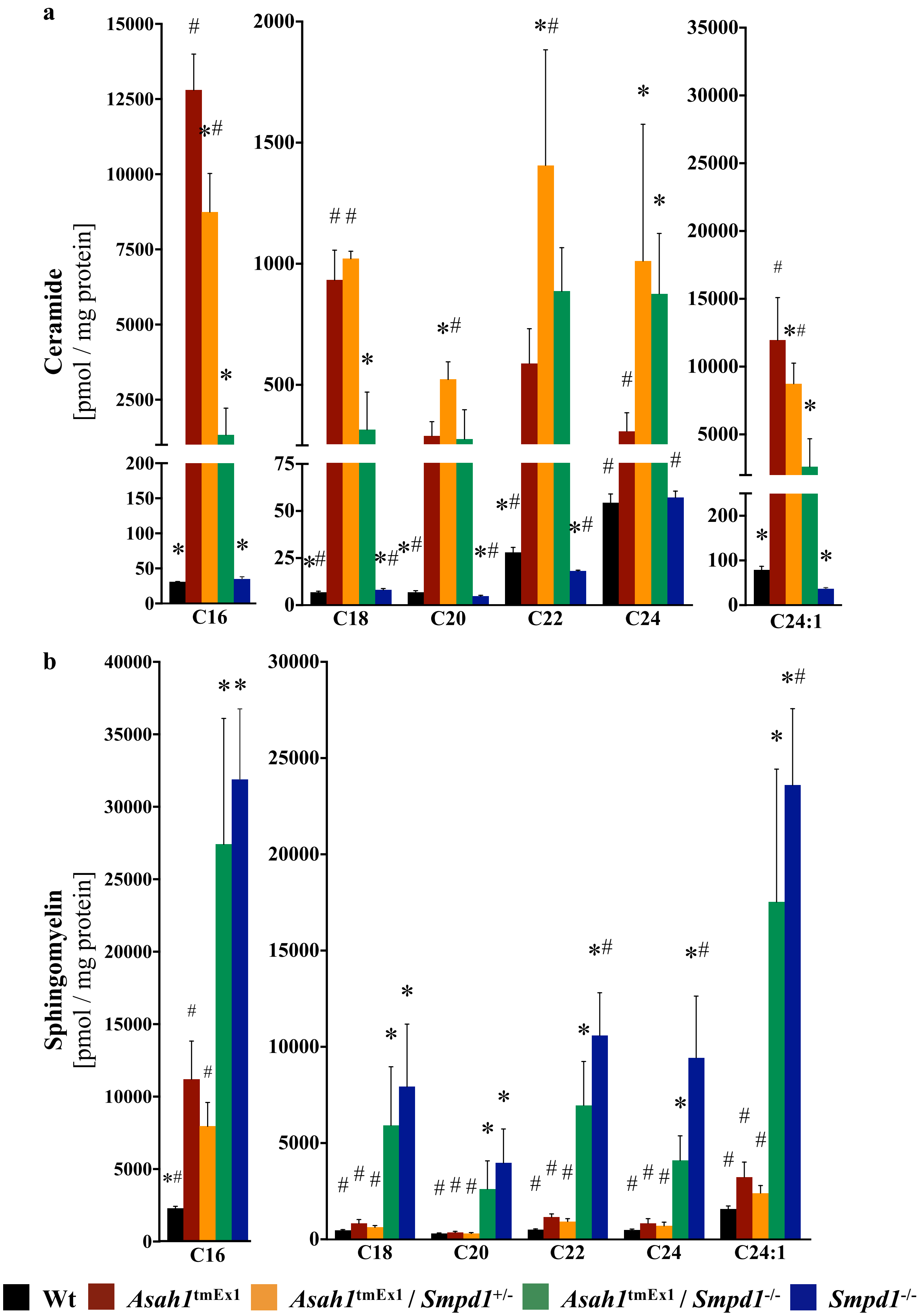

Supplementary Figure 1. Accumulation of different ceramide and sphingomyelin species in acid ceramidase deficient mice and effect of acid sphingomyelinase co-ablation. (**a-b**) The effects of Asm co-deficiency on ceramides (**a**) and sphingomyelins (**b**) of different chain lengths were analyzed by liquid chromatography tandem-mass spectrometry (LC-MS/MS) of snap-frozen spleen samples of Ac deficient mice. * $p < 0.05$ compared to *Asah1*$^{\text{tmEx1}}$, # $p < 0.05$ compared to *Asah1*$^{\text{tmEx1}}$ / *Smpd1*$^{-/-}$ (ANOVA with Dunnett posttests). The legend at the bottom of the figure applies to both (**a**) and (**b**).
