# Supplementary material for: Acid Sphingomyelinase Deficiency Ameliorates Farber Disease
Source: Int J Mol Sci. 2019 Dec 11;20(24):6253. doi: 10.3390/ijms20246253 (PMC6941101; doi:10.3390/ijms20246253)
Supplement: Supplementary file 1 [file ijms-20-06253-s001.zip › ijms-637307-supplementary/suppl figure 2.pdf]

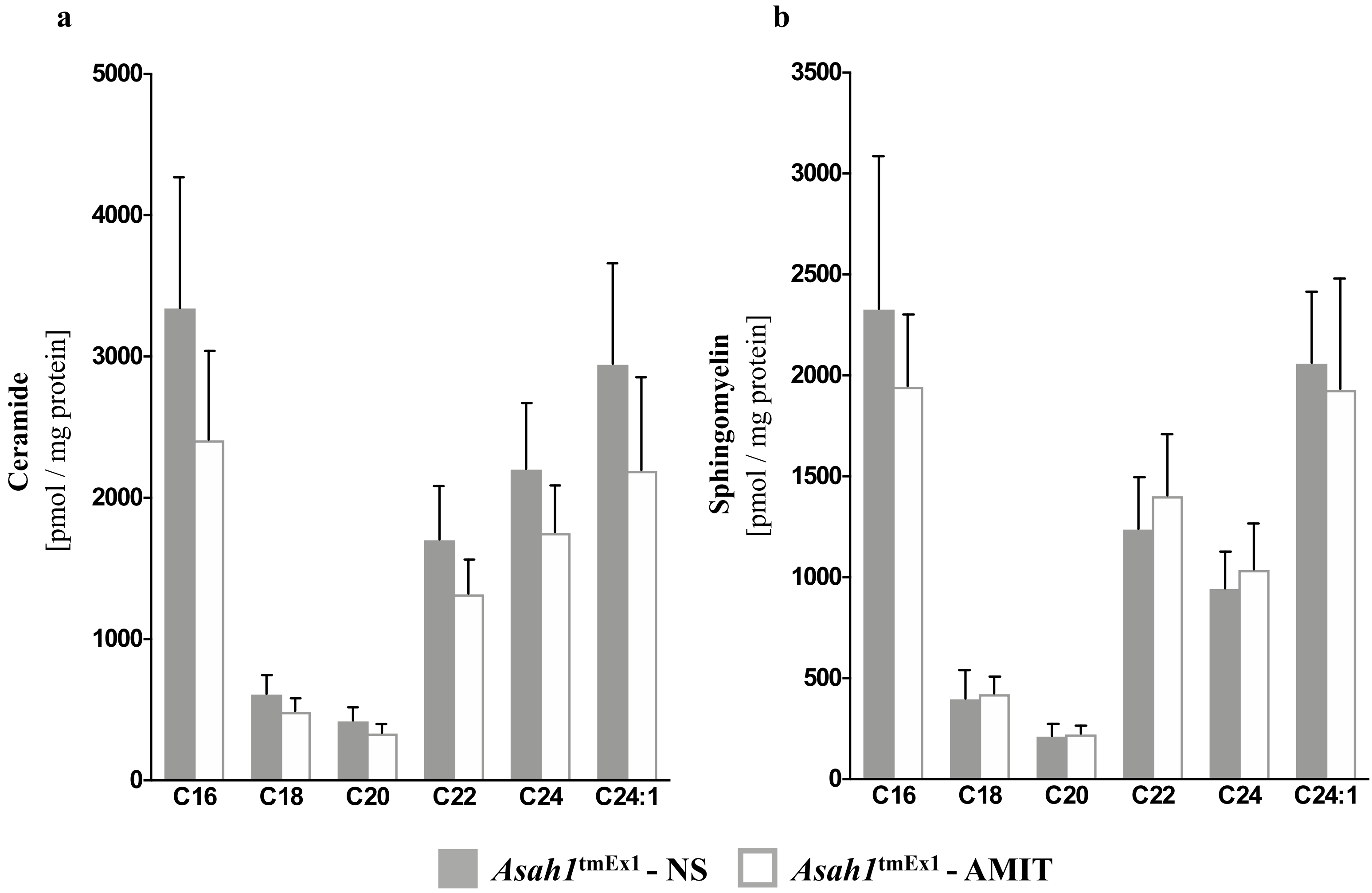

Supplementary Figure 2. Effect of amitriptyline treatment on different ceramide and sphingomyelin species in acid ceramidase deficient mice. (**a-b**) The effects of amitriptyline treatment on ceramides (**a**) and sphingomyelins (**b**) of different chain lengths were analyzed by liquid chromatography tandem-mass spectrometry (LC-MS/MS) of snap-frozen spleen samples of Ac deficient mice. n = 3-5 mice/group. No significant differences were detected compared to *Asah1*^{tmEx1} - NS (Student t test). The legend at the bottom of the figure applies to both (**a**) and (**b**).
